# Exploration of flipped classroom approach to enhance critical thinking skills

**DOI:** 10.1016/j.heliyon.2023.e20895

**Published:** 2023-10-14

**Authors:** Yan Ma

**Affiliations:** Department of Business Administration, XIANDA College of Economics & Humanities Shanghai International Studies University, 200083, Shanghai, China

**Keywords:** Flipped classroom approach, Critical thinking skills, Business, *Marketing*

## Abstract

Flipped classroom approach has become well-accepted and widely-used in different levels of education. Critical thinking was deemed as the core goal of higher education institutions to enhance students' competence advocated in the 21st century. The definition of critical thinking originating from Peter Facione was one of the most widely-accepted and commonly-used at present. The aim of the research was to explore a flipped classroom approach which was effective to enhance critical thinking skills proposed by Peter Facione, namely interpretation, analysis, inference, evaluation, explanation and self-regulation. This study selected 300 junior students majoring in business administration (100 students), international trade (100 students), and accounting (100 students) respectively of a private university in China, and took the *Marketing* flipped classroom course as an example. The quantitative research method was adopted in the research, and a questionnaire with 22 questions was used to collect data from the participants. The study involved two rounds. Each round involved three control classes with a traditional teaching instruction approach conducted and three experimental classes with a flipped classroom approach adopted. After analyzing and discussing the data collected, the research found that the flipped classroom approach promoted students' critical thinking skills to some extent. Through the study, a modified model of flipped classroom approach to enhance critical thinking skills was put forward. The modified model can be applied to more other courses at different levels, especially business courses. The research also had some limitations, including the 5-point Likert scale used, the representativeness and individual differences of participants, and the application of the study. In the future research, aspects regarding other skill development, effectiveness of course feedback and assessment, interdisciplinary knowledge, and implementation of the marketing plan etc.can be explored deeper.

## 1 Introduction

Flipped classroom approach is an inverted learner-centered pedagogical model. The simplest definition for this model is: what is normally done during class time is shifted to home activities and what is usually done at home will be transferred to as class activities. Accordingly, students watch videos on course contents at home and present their learning outcomes in class. With the development of internet technology and digital media, flipped classroom approach, originating from two high school teachers named Jonathan Bergmann and Aaron Sams in 2007, has become well-accepted and widely-used in different levels of education [1].

Several studies [[2], [3], [4], [5], [6], [7], [8]] have confirmed that flipped classes really exerted more positive impacts on students' learning performances and outcomes, autonomous learning motivation, self-efficacy, knowledge understanding and applying, teacher-student interaction, group or peer interaction, communication, teamwork, time management, and creativity abilities than traditional lectures. Students were more likely to be satisfied with the learning experience and process in flipped classes, and could achieve better learning performance and academic results compared with traditional classroom learning [9].

In recent years, many researchers in business fields implemented flipped classroom approach by blending with a model or a tool to compare the learning effects or skill development among undergraduate students with traditional learning approach. Huang et al. [10] introduced a flipped classroom linking with 'Business Simulation Games' (BSGs) which was a skill development tool, and proved that the use of BSGs really had influences on improving students' critical thinking. Durrani et al. [11] studied undergraduate students who majored in management and IT. The study combined flipped classroom with gamification learning approach, and found that the gamified flipped classroom was an effective methodology which could better develop undergraduate students' overall skills, including technique complexity, task orientation clarity, student engagement, overall students' satisfaction, and learning motivation, than adopting a traditional way of teaching. Zainuddin [12] also found that the gamified flipped-class instructions had positive correlations with students' competence, learning autonomy and motivation, class performance and engagement, as well as learning achievement and outcomes. A gamified flipped learning could also foster students' competences in writing and presentation skills, as well as comprehension [13]. Apart from the blending studies, some researchers focused on the instructors' attitudes toward the learning outcomes of undergraduate management students and confirmed the positive correlation between them in the flipped classes [14]. Other researchers proved that flipped classroom approach could improve the performances of undergraduate business students with early classes starting at 8:00 a.m., and also help those students whose classes begin at 9:00 a.m. by taking a nap at noon [15].

From another side, in order to cultivate talents with innovative thinking quality and international vision, colleges and universities are focusing on cultivating students' critical thinking abilities. Critical thinking, a crucial higher-order thinking skill, was regarded as the core goal of higher education institutions to enhance students' competence advocated in the 21st century. It's essential to cultivate students' critical thinking, and therefore significantly facilitating students' academic performance and thinking development, as well as problem-solving skills [16,17]. There are many definitions of critical thinking. Nowadays, one of the most widely-accepted and commonly-used definitions was from Peter Facione [18] who once stated that 'we understand critical thinking to be purposeful, self-regulatory judgement which results in interpretation, analysis, evaluation, and inference, as well as explanation of the evidential, conceptual, methodological, criteriological, or contextual considerations upon which that judgement is based'. Dong [19] adapted the six critical thinking skills, namely interpretation, analysis, inference, evaluation, explanation, and self-regulation, on the basis of Facione's research. Interpretation is to understand the concept and express its meaning. Analysis is to distinguish the internal relationship and logical reasoning relationship of various elements in the concept. Inference means seeking and questioning evidences, constructing and considering multiple possibilities. Evaluation is to make a decision after comparing the credibility, relevance and advantages of various views. Explanation is to explain the results comprehensively and clearly, and defend the reasoning process. Self-regulation means self-reflection, inspection and correction of metacognition.

Regarding the blending researches on flipped classroom approach and critical thinking skills, many researchers conducted those studies in the fields of nursing or health sciences. Different researchers got different findings. In some researches [20,21], flipped classroom was proved to be an effective methodology which could develop undergraduate students' creative and critical thinking skills and social awareness, as well as obtain better results than adopting a traditional way of teaching. However, Sezer and Esenay [22] compared undergraduate nursing students who followed an online traditional learning approach with those who followed a flipped classroom learning approach, and found that there were no big differences on students' learning achievements and critical thinking skills by adopting the two learning approaches.

There are two research gaps in the existing literature. On the one hand, although numerous studies have investigated flipped-class instructions, little research has been done on the combination of flipped classroom approach and critical thinking skills in business fields. On the other hand, many previous researches approved of the importance of critical thinking skills and accepted Peter Facione's definition, while few studies researched on how flipped classroom approach influenced the enhancement of specific skills in the definition.

Accordingly, this study sought to answer the following two research questions.

RQ1 What activities can be used in the flipped classroom approach to enhance students' critical thinking skills?

RQ2 Whether the modified model of flipped classroom approach has impacts on enhancing students' critical thinking skills?

If the two research questions can be addressed, more researchers and students can benefit from this study. For researchers, more inspirations can be aroused and further studies may be conducted regarding the educational approaches improving specific skills or abilities, or the implications of the study in other courses, especially business courses. For students, if students can adopt the appropriate learning methods, students' learning efficiency will be enhanced and learning interests will be motivated.

The paper has seven sections. Section 1 introduces literature on related studies in flipped classroom approach and background of critical thinking skills. Section 2 provides materials and methods of the research, including participants, instruments, designs, and procedures. In section 3, data collected for the questionnaires are compared and analyzed. Section 4 discusses the four main findings related to the two research questions based on the results in section 3. Section 5 explores the practical implication of the study and explains the theoretical contribution. Section 6 concludes with the research questions, and section 7 puts forward the research's limitations, pedagogical implications and directions for future researches.

## 2 Materials and methods

### 2.1 Participants

The data was collected from 300 students in a Chinese private university. The participants were all junior students majored in business administration (100 students), international trade (100 students), and accounting (100 students) respectively, since the three major students should learn *Marketing* course in the business school of the university. It took each student one semester to learn *Marketing* course. Each semester had 16 teaching weeks and two examination weeks. Each teaching week had three seminars (45 min per seminar). The study was conducted two rounds, with 150 students each semester. The classes were randomly divided, and the degree of students in the two classes of each major was similar.

As shown in Table 1, this study divided the two classes of students in each major into a control class and an experimental class, which meant that each round had a control class (25 students) and an experimental class (25 students) in the business administration major, a control class (25 students) and an experimental class (25 students) in the international trade major, and a control class (25 students) and an experimental class (25 students) in the accounting major.

**Table 1:** Participant majors and numbers.

**Major**	**Number**			
	**The first practice**		**The second practice**	
	**Control classes (75 students totally)**	**Experimental classes** **(75 students totally)**	**Control classes (75 students totally)**	**Experimental classes** **(75 students totally)**
**Business Administration**	25	25	25	25
**International Trade**	25	25	25	25
**Accounting**	25	25	25	25

### 2.2 Instruments

**Fig. 1:**
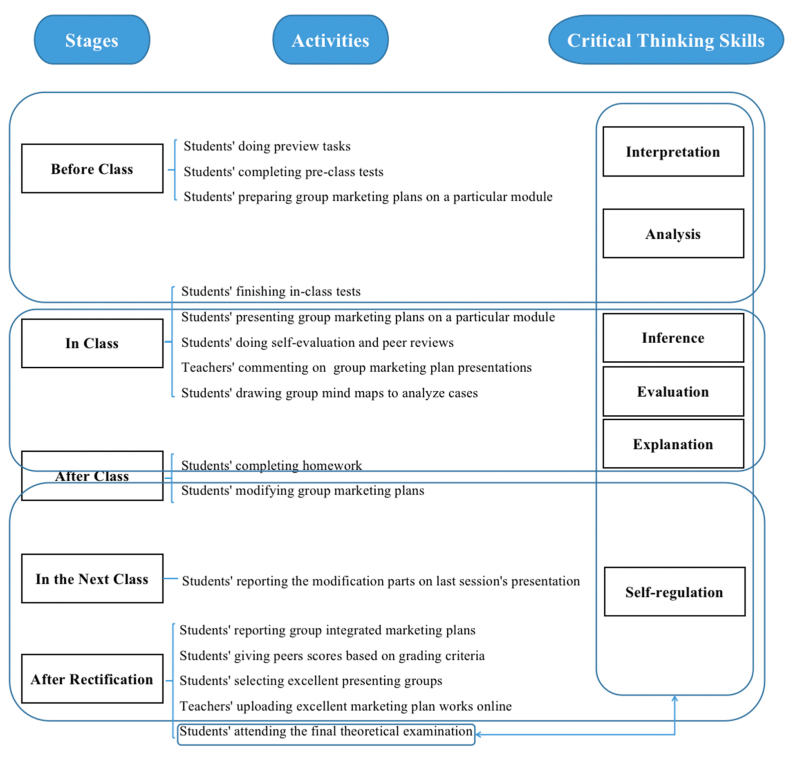
Model of activities used in the flipped classroom approach to enhance critical thinking skills.

The study adopted a quantitative research method by using a questionnaire. In order to know whether hypothesized model of flipped classroom approach (Fig. 1) in this study was effective, a questionnaire (See Appendix 1) with 25 questions was designed to collect students' feedback. The 25 questions could be segmented into five parts which were students' preferences for learning styles, students' feedback on preview tasks before class, students' feedback on presentation and peer evaluation in class, students' reflection and evaluation feedback after class, and a multiple choice regarding students' satisfaction factors with the course. Before the questionnaire was sent to the participants, a pilot was carried out among 20 students selected at random. After the pilot, two places were modified. Firstly, for the students' preferences for learning styles, a 5-point Likert scale on four statements was changed into a single choice question, since a student might agree that he liked all the learning styles, the data collected might be invalid. Secondly, the multiple choice on students' satisfaction with the course was changed into an open question on students' satisfaction or dissatisfaction with the course, for students might have more opinions, both positive or negative, on the course. In addition, the guidance was added to the open question because students might be confused on what aspects to state. The guidance made students clear that they could refer to the teaching method, task requirements and varieties, online and offline resources, in-class activities, homework load, evaluation and assessment, teaching feedback and guidance, class atmosphere, and interaction between the teacher and students, etc. Therefore, the revised questionnaire (See Appendix 2) involved 22 questions, with one single choice on students' preferences for learning styles, a 5-point Likert scale on 20 statements related to students' feedback on the flipped classroom approach, and one open question on what aspects students satisfied or dissatisfied with the course. The revised questionnaire was sent to all the control and experimental classes online to collect their feedback on the *Marketing* course at the end of the semester. The reason for choosing the online questionnaire was that it was a fast and effective way of collecting large amounts of data and information from a large number of respondents.

**Fig. 2:**
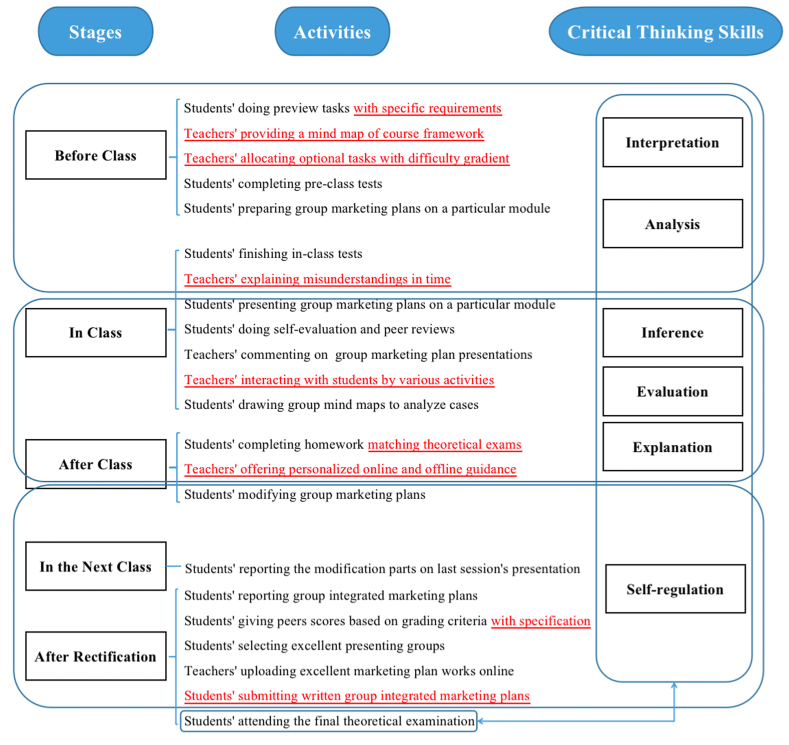
Modified model of activities used in the flipped classroom approach to enhance critical thinking skills.

All respondents' feedback on whether classroom activities in flipped classrooms were effective for enhancing their critical thinking abilities (Fig. 1) in the first practice could contribute to revising the model of activities used in the flipped classroom approach (Fig. 2) in the second practice. In the modified model, three more factors were taken into consideration before class. Firstly, preview tasks should have specific requirements. Secondly, the teacher should provide a concrete mind map of framework on each module's key and difficult points online, which could guide students to learn. Thirdly, the teacher should consider students' individual differences and different learning abilities, and thus allocating optional tasks with difficulty gradient. In class, timely guidance for students' misunderstandings in the in-class tests, and interaction with students by various activities should be implemented. After class, four factors should be added to the modified model. First of all, homework question types could adopt theoretical concept selection and judgment, short essay questions, discussion questions, and case analysis, since they matched the question types in the final theoretical examination, which could check students' enhancement of overall critical thinking skills. Secondly, more teacher's personalized online and offline tutoring and guidance were required. Thirdly, grading criteria should be specified. Last but not least, a written group marketing plan based on the modified group integrated marketing plan presentation should be added to give students chances to modify the group marketing plan.

In order to know whether modified model of flipped classroom approach had influences on enhancing critical thinking skills, group integrated marketing plan paper results and theoretical examination results were compared to assess the enhancement influences. Both control classes and experimental classes should report group integrated marketing plans, submit written group integrated marketing plans at the last teaching seminar, and fulfill the theoretical examination paper at the examination weeks.

The reason for requiring both oral and written group integrated marketing plan was to check students' overall understanding of the theories and applying abilities. The group integrated marketing plan could show students' understanding of the course contents, potential of logical analysis and inference, capabilities for comprehensively explaining and evaluating. Since the written group integrated marketing plan was based on the class group marketing plan presentation in which the modification suggestions were given by the peers and the teacher, the teacher could check whether students corrected their wrong understandings and improved the works. All the factors involved in the group integrated marketing plans could reflect students' six critical thinking skills.

The reason why the theoretical exams were chosen was that the theoretical exams mainly involved theoretical concept selection and judgment, short essay questions, discussion questions, and case analysis. Students could choose or judge the theoretical concepts based on their understanding of the marketing theories and definitions, which could test their 'interpretation' skills. After students had understood the short essay questions and discussion questions, they could analyze the elements of the questions, find evidences and multiple possibilities to answer the questions, make a decision on the best answer, and finally explain the reasons for the answer comprehensively, thus proving students' 'analysis', 'inference', 'evaluation' and 'explanation' skills. Apart from all the five above critical thinking skills, case analysis required students' reflection on what they knew and comprehensively applied to the new case context, which required students' 'self-regulation' skills.

### 2.3 Designs

The study involved two rounds. The first round was conducted in the autumn semester from 2020 to 2021, while the second round was carried out in the autumn semester from 2021 to 2022. Each round involved three control classes and three experimental classes from business administration, international trade, and accounting majors respectively, with 25 students each class. All the courses in two rounds were taught by the same teacher. The control classes conducted a traditional teaching instruction approach while the experimental classes carried out a flipped classroom approach.

There were some similarities between control class learning and experimental class learning. Preview tasks and pre-class tests were required for both control classes and experimental classes. In class, the teacher checked students' self-learning by in-class tests and explained the contents that students did not understand by more cases or exercises. After class, students completed assignment such as theoretical concept selection and judgment, short essay questions, discussion questions, and case analysis, which matched the question types in the final theoretical examination. This course had 16 teaching weeks. 14 modules were taught and finished in the 14 teaching weeks. The contents of each module was integrated into the situation of a self-founded company, and a group presentation of marketing plan was made. According to different abilities, students could choose between creating a new brand or imitating an existing brand. Both control class and experimental class students were required to report on the group integrated marketing plan in the last two teaching weeks. After each group's presentation, the presenting groups would do a self-evaluation, and received peer reviews and the teacher's comments. Then, other audience groups compared the advantages and disadvantages of various views, and combined the grading criteria to give the presenting group a score on the spot. After the peer evaluation, students would also select the excellent groups on the spot. Furthermore, each presenting group should improve the marketing plan based on peers' and the teacher's feedback, and finally submitted the revised group integrated marketing plan in the written form at the last teaching seminar. Then, the teacher would upload the excellent marketing plan works on the online platform. At the examination weeks, students in both control classes and experimental classes should take the final theoretical examination.

The differences between control class learning and experimental class learning were as follows. For control classes, the teacher gave lectures on course contents and interacted with students in class, while for experimental classes, the teacher adopted the flipped classroom approach shown in Fig. 1 in the first practice and taught the modified model shown in Fig. 2 in the second practice. For experimental groups, at the beginning of the course, the teacher assigned an integrated marketing plan task which required students preview course contents before class and discuss in the groups to make a part of the marketing plan based on the module previewed each week. In class, students gave presentations in groups on the marketing plan, and then, self evaluation, peer reviews, and the teacher's comments were conducted on each presenting groups. In this way, the teacher did not teach course contents in class, but the teacher would know what misunderstandings students had according to their presentations. In order to eliminate students' misunderstandings, the teacher provided comments on the misunderstandings, illustrated with more cases, led students to analyze cases by drawing mind maps, assigned more exercises or activities to consolidate students' understanding and application, etc. After class, students should modify the group marketing plan based on peer and the teacher's comments, and reported the rectifications in the next class.

### 2.4 Procedures

In the first practice, all the experimental classes adopted the flipped classroom approach shown in Fig. 1 while all the control classes adopted a traditional learning approach. At the end of the class, a questionnaire (See Appendix 2) after the pilot was sent to all the participants online. It would take participants about 5 min to fulfill the questionnaire. Participants were required to answer the online questionnaire in one week, and they were allowed to answer the questionnaire at any time. Since *Marketing* course was a model course to conduct flipped classroom approach in the author's university, the study was supported and approved by the university. This research carried out an ethical approval of the university and informed consents were obtained from all the participants for the research. All the participants had been provided with relevant information about the study. Data was confidential, with no personal information stored. Participants were voluntary, and full anonymity was guaranteed. Students in both control classes and experimental classes would finish the course contents in 14 teaching weeks, conducted the group marketing plan presentations in 15–16 teaching weeks, and participated in the final theoretical examination in 17–18 examination weeks.

After the first practice, the teacher modified the model of the flipped classroom approach based on the analysis of students' feedback on the questionnaire. A modified model of activities used in the flipped classroom approach (Fig. 2) was carried out in three experimental classes in the second practice while all the three control classes still adopted a traditional learning approach. The second round of the teaching practice adopted the same procedures as the first practice, and a questionnaire was sent and the feedback was collected as well at the end of the second practice. A comparison was made based on the questionnaire feedback, results of group integrated marketing plans, and academic results of the final theoretical examinations according to two rounds' data.

**Fig. 3:**
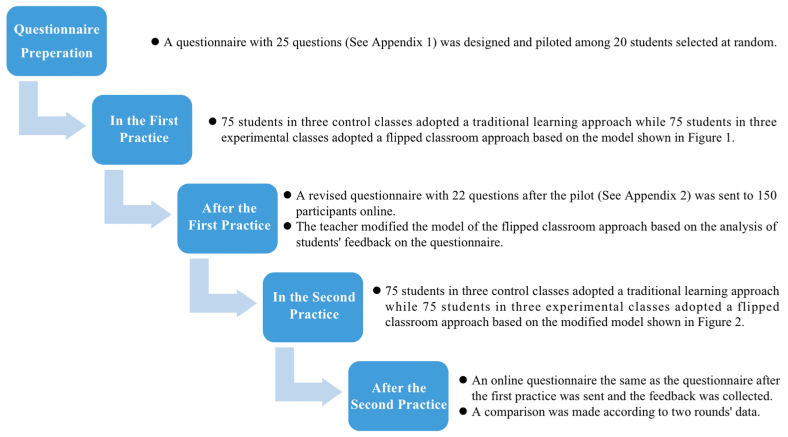
The framework of the study.

The framework of the study was concluded as Fig. 3 shown.

## 3 Results

300 online questionnaires were sent to the participants, and all the 300 feedback on the questionnaires was collected online, with a 100 % response rate.

The 22 questions in the questionnaire (See Appendix 2) could be divided into five parts, which were students' preferences for learning styles, students' feedback on preview tasks before class, students' feedback on presentation and peer evaluation in class, students' reflection and evaluation feedback after class, and students' satisfaction and dissatisfaction with the course. A single choice question was designed for knowing students' preferences for learning styles. A 5-point Likert scale with 20 statements were designed to collect students' feedback on preview tasks before class, students' feedback on presentation and peer evaluation in class, and students' reflection and evaluation feedback after class. Participants were asked to read 20 statements and match their thoughts with a number from 1 to 5 (1 means 'Strongly Disagree', 2 means 'Disagree', 3 means 'Neutral', 4 means 'Agree', and 5 means 'Strongly Agree'). An open question was designed to discover students' satisfaction and dissatisfaction with the course further.

### 3.1 Students' preferences for learning styles

Students were asked to choose one of the favorite learning styles among self-learning with tests checking effects, self-learning with presentation and evaluation, the teacher' guidance with the flipped classroom approach, and the traditional teaching instruction approach. Table 2 illustrated students' preferences for learning styles. In general, for control classes, 85.33 % respondents (64 students) after the first practice and 88 % respondents (66 students) after the second practice would like to accept the traditional way (Choice D) of learning in which the teacher gave lectures in class and students learned. Control groups were quite familiar with the traditional teaching instruction approach and might not try other teaching approaches, that was why most students chose this teacher-dominated way.

**Table 2:** Students' preferences for learning styles.

**Statements**	**Number (%)**			
	**The first practice**		**The second practice**	
	**Control classes (75 students totally)**	**Experimental classes (75 students totally)**	**Control classes (75 students totally)**	**Experimental classes (75 students totally)**
A. I like self-learning first, and then the teacher tests the learning effects in class. The teacher just explains the problems reflected on the tests without teaching the course contents.	1 (1.33)	2 (2.67)	3 (4.00)	1 (1.33)
B. I like self-learning by finishing the preview tasks, and then present the preview effects in class. The peers and the teacher comments on the preview tasks. The teacher explains the problems reflected on the students' preview tasks without teaching the course contents.	2 (2.67)	17 (22.67)	1 (1.33)	38 (50.67)
C. I hope that the teacher will explain and guide me before I finish my preview tasks, and then present the preview effects in class. The peers and the teacher comments on the preview tasks. The teacher explains the problems reflected on the students' preview tasks without teaching the course contents.	8 (10.67)	38 (50.67)	5 (6.67)	25 (33.33)
D. I like the traditional way of learning. The teacher gives lectures in class and I learn instead of finishing the preview tasks and presenting the works in class.	64 (85.33)	18 (24.00)	66 (88.00)	11 (14.67)

For experimental classes, after the first practice, only 17 students (22.67 %) favored the way (Choice B) that 'students did self-learning by finishing the preview tasks, and then presented the preview effects in class. The peers and the teacher commented on the preview tasks. The teacher explained the problems reflected on the students' preview tasks without teaching the course contents'. This way matched the flipped classroom teaching model shown in Fig. 1. However, 38 students (50.67 %) hoped that the teacher could give them some guidance before they adopted this flipped classroom approach (Choice C). After the second practice which adopted a modified model shown in Fig. 2, more than half of the students (38 students) were willing to accept the flipped classroom approach (Choice B) while a third of the cohorts (25 students) wanted some teacher's guidance before their self-learning (Choice C). In both practices, very few students would like to choose the mainly self-learning way (Choice A). This result indicated that students had individual differences and different learning abilities, so they would choose different learning styles.

### 3.2 Students' feedback on preview tasks before class

There were six statements related to students' feedback on preview tasks before class. According to Table 3, three interesting phenomena could be found.

**Table 3:**
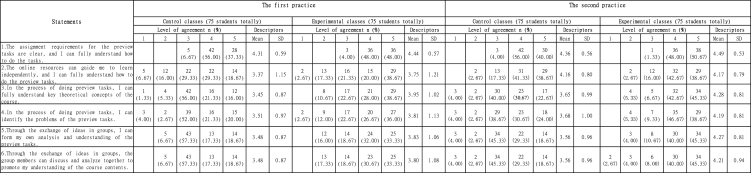
Students' feedback on preview tasks before class.

Firstly, after the first practice, control classes and experimental classes had 17 (5 + 12) and 15 (2 + 13) students, 22.67 % and 20 % participants respectively, disagreed or strongly disagreed that the online resources could guide them to learn independently, and they might not fully understand how to do the preview tasks (Statement 2) though 70 (42 + 28) respondents in control classes and 72 (36 + 36) respondents in experimental classes approved of the clearness of the assignment requirements (Statement 1). Compared with the first practice, over 80 % of the students in both cohorts including 60 (31 + 29) students in control classes and 61 (32 + 29) students in experimental classes agreed or strongly agreed that the online resources did guide them to understand the preview tasks to a certain extent after the second practice (Statement 2).

Secondly, based on statements 3–6, in terms of the process of doing preview tasks, statistics on both practices indicated that control classes had more uncertainties (more participants chose 'Neutral') about preview tasks than experimental classes, whether it was for individual understanding of key theories and identification of the problems, or enhancement of understanding and analysis abilities through group discussions and exchanges.

Thirdly, according to statements 3–6, still about 20 % of the respondents (14–17 students) in experimental classes selected 'Neutral' for most of the statements after the first practice, which meant that they were not so sure about their understanding and analysis improvement through preview tasks. After the second practice, over 85 % participants (64–66 students) agreed or strongly agreed that this flipped classroom approach could help them understand the curriculum framework and knowledge in the preview stage, clarify key theoretical concepts, and form their own understanding and analysis of preview task problems through communication in the process of completing preview tasks with group members.

### 3.3 Students' feedback on presentation and peer evaluation in class

There were six statements related to students' feedback on presentation and peer evaluation in class. Through the comparison between two practices in Table 4, three main results could be concluded as follows.

**Table 4:**
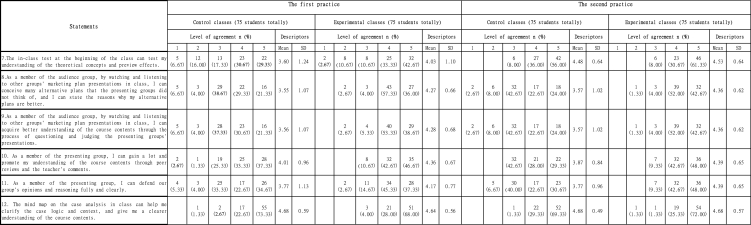
Students' feedback on presentation and peer evaluation in class.

In terms of statement 7 which related to in-class tests, after the first practice, only 60 % respondents (23 + 22 = 45 students) in control classes and 76 % respondents (25 + 32 = 57 students) in experimental classes agreed or strongly agreed that in-class tests at the beginning of the class could test their understanding of the theoretical concepts and preview effects. Other 17 (12 + 5) students in control classes and 10 (8 + 2) students in experimental classes disagreed or strongly disagreed with the in-class test statement. However, after the second practice, 92 % respondents (27 + 42 = 69/23 + 46 = 69 students) approved or strongly approved of the effectiveness of the in-class tests in both cohorts after the second practice.

As for the statements 8–11 regarding the group integrated marketing plan presentation, both first and second practices showed that generally experimental groups achieved a higher sense of identity for the enhancement of several critical thinking abilities through the process of group marketing plan presentation than control groups did.

Statement 12 regarding the mind map which ranked the highest identities among statements 7–12 in the in-class questionnaire part, more than 96 % respondents (72–74 students) of all groups in both first and second practices agreed or strongly agreed that the mind map on the case analysis in class could help them clarify the case logic and context, and give them a clearer understanding of the course contents.

### 3.4 Students' reflection and evaluation feedback after class

Based on Table 5, four major conclusions could be drawn from the eight statements regarding students' reflection and evaluation feedback after class.

**Table 5:**
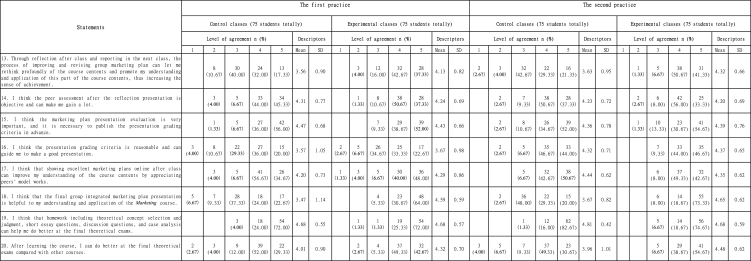
Students' reflection and evaluation feedback after class.

First of all, according to statement 13, after the first practice, 60 (32 + 28) respondents in experimental groups agreed or strongly agreed that they could rethink profoundly of the course contents and promote the understanding and application abilities through reflection on the group marketing plan after class and reporting in the next class, while only 37 (24 + 13) respondents in control groups approved or strongly approved of the statement. After the second practice, 69 (38 + 31) respondents in experimental groups approved or strongly approved of the above statement while only 38 (22 + 16) respondents in control groups agreed or strongly agreed.

Secondly, based on statement 14, 15 & 17, over 85 % of all groups (64–70 students) in both practices approved or strongly approved of the objectiveness and importance of peer assessment based on the sound grading criteria, as well as the appreciation of peers' model works. However, according to statement 16, only 56 % respondents (27 + 15 = 42/25 + 17 = 42 students) in control and experimental classes after the first practice considered the presentation grading criteria as reasonable. Since the original grading criteria in the model (Fig. 1) were quite general, including contents, delivery, teamwork and overall evaluation, accounting for 25 % each category, which could not lead students to make a better marketing plan presentation. The improved grading criteria specified contents, delivery, teamwork and overall evaluation for the second practice. Compared with the first practice, Table 5 showed the good effects after the second practice, with 68 (35 + 33 = 68/33 + 35 = 68) students in both cohorts, that was 90.67 % respondents, considering grading criteria as reasonable (Statement 16).

Thirdly, refer to statement 18, for the first practice, the number of respondents (18 + 17 = 35 students) in control groups who agreed or strongly agreed that the final group integrated marketing plan was helpful for the understanding and application of the *Marketing* course was less than half of the number (23 + 48 = 71 students) in experimental groups. For the second practice, there was still a significant number gap on the statement agreement between control groups (22 + 15 = 37 students) and experimental classes (14 + 55 = 69 students). That might result from the reason that control groups were not as good at internalizing theories from marketing plan presentation as experimental groups did as mentioned in marketing plan presentation reflection part.

Last but not least, based on the data of homework allocation validity and final theoretical examination confidence in statements 19 & 20, each group in both practices had more than 70 out of 75 respondents approving or strongly approving of the homework allocation validity by agreeing the statement that homework including theoretical concept selection and judgment, short essay questions, discussion questions, and case analysis could help them do better at the final theoretical exams (Statement 19). Generally, participants in experimental groups for both practices were more confident in their final theoretical examination than those in control groups (Statement 20). After the first practice, 61 (39 + 22) respondents in control classes and 69 (37 + 32) respondents in experimental classes respectively thought that they could do better at the final theoretical exams compared with other courses after learning the *Marketing* course. After the second practice, 60 (37 + 23) control group students and 70 (29 + 41) experimental group students approved or strongly approved of the statement 20.

### 3.5 Students' satisfaction and dissatisfaction with the course

Based on Table 6, after the first practice, students involved in control and experimental groups were satisfied with mainly nine factors, including the teacher's personality (82 students mentioned), the teacher's teaching method (67 students mentioned), class atmosphere (65 students mentioned), interaction between the teacher and students (58 students mentioned), varieties of tasks and assignments (43 students mentioned), course assessment (27 students mentioned), mind map used (22 students mentioned), the teacher's offline guidance (9 students mentioned) and homework load (8 students mentioned).

Surprisingly, after the second practice, students in both cohorts were still mainly satisfied with the above nine factors. According to Table 6, 99 students mentioned the teacher's personality, 90 students mentioned the teacher' teaching method, 77 students mentioned class atmosphere, 76 students mentioned interaction between the teacher and students, 65 students mentioned varieties of tasks and assignments, 26 students mentioned course assessment, 24 students mentioned mind map used, 15 students mentioned the teacher's offline guidance, and 7 students mentioned homework load.

**Table 6:** Students' satisfaction with the course.

**Satisfaction factors**	**Number**					
	**The first practice**			**The second practice**		
	**Control classes (75 students totally)**	**Experimental classes** **(75 students totally)**	**Total number (150 students totally)**	**Control classes (75 students totally)**	**Experimental classes** **(75 students totally)**	**Total number (150 students totally)**
**The teacher's personality**	40	42	82	50	49	99
**The teacher's teaching method**	22	45	67	34	56	90
**Class atmosphere**	17	48	65	25	52	77
**Interaction between the teacher and students**	10	48	58	34	42	76
**Varieties of tasks and assignments**	6	37	43	18	47	65
**Course assessment**	18	9	27	18	8	26
**Mind map used**	9	13	22	6	18	24
**The teacher's offline guidance**		9	9		15	15
**Homework load**	8		8	5	2	7

According to the statistics in Table 6, more students in experimental classes were satisfied with the teacher's teaching method, class atmosphere, interaction between the teacher and students, varieties of tasks and assignments especially the mind map used, and the teacher's offline guidance than those in control classes did after both practices. This finding showed that flipped classroom method was effective to some extent, since flipped classroom method required more interaction, task varieties, and the teacher's guidance than traditional teacher-dominated way. However, more students in control classes were content with course assessment and homework load. Those students stated that they favored homework matched course assessment tightly, which might contribute to a high mark in the final theoretical examination. Since Chinese students focused on scores in the final theoretical examination, and the assessment in the form of theoretical examination was quite favorable among those students.

From students' point of view, whether in control classes or in experimental classes, they liked kind teachers who could use new ways of teaching, guide students patiently, explain course contents clearly, create a sound class atmosphere which involved adequate interaction between the teacher and students, allocate various tasks and assignments with clear requirements and reasonable workload, and assign homework related to course assessment, etc.

Table 7 illustrated seven main dissatisfaction factors raised by all participants. According to 150 students participated in the first practice, the seven major dissatisfaction factors involved homework load (13 students mentioned), higher requirements for students' abilities (8 students mentioned), interaction between the teacher and students (8 students mentioned), course assessment (6 students mentioned), group work (6 students mentioned), no opportunities to revise the marketing plan (6 students mentioned), and lacking varieties of tasks and assignments (4 students mentioned). In those experimental students' opinions, flipped classroom approach required more homework load and had higher requirements for students' abilities, which put pressure on the students with low level of abilities. In those control groups' view point, interaction between the teacher and students were inadequate, and tasks lacked of varieties. They also stated that there were no opportunities to revise marketing plan at the last two seminars. Moreover, several students didn't like group work and course assessment, and therefore, more teacher's personalized online and offline tutoring and guidance were required.

**Table 7:** Students' dissatisfaction with the course.

Dissatisfaction factors	Number					
	The first practice			The second practice		
	Control classes (75 students totally)	Experimental classes (75 students totally)	Total number (150 students totally)	Control classes (75 students totally)	Experimental classes (75 students totally)	**Total number (150 students totally)**
Homework load	3	10	13	1	4	5
Higher requirements for students' abilities		8	8		3	3
Interaction between the teacher and students	5	3	8	3		3
Course assessment	2	4	6	1	2	3
Group work	3	3	6	1	2	3
No opportunities to revise the marketing plan	6		6			
Lacking varieties of tasks and assignments	3	1	4			

After conducting the modified flipped classroom approach (Fig. 2) after the second practice, fewer students raised these dissatisfaction factors. After the second practice, among both cohorts, only 5 students mentioned homework load, 3 students mentioned higher requirements for students' abilities, interaction between the teacher and students, course assessment, and group work respectively. No students mentioned that there were no opportunities to revise the marketing plan or the course lacked varieties of tasks and assignments. The above results proved that the modified model was effective to a certain extent.

## 4 Discussion

Based on the results in section 3, four main findings related to the two research questions were discussed further as follows.

### 4.1 Preview tasks before class improving students' 'interpretation' and 'analysis' abilities

Due to the differences in students' individual learning habits and self-learning abilities, the same tasks might not be fit for all the students. Lai et al. [23] once stated that flipped classroom asked higher requirements for both learners and teachers. The effectiveness of flipped classroom was closely related to students' personal abilities. If students' self-efficacy was high, students would acquire higher autonomous learning motivation and participate in discussions more actively. Therefore, in this study, the teacher took task difficulty gradient and optional tasks into account in the modified model (Fig. 2). For example, when designing the group marketing plan, students could choose between creating a new brand or imitating an existing brand.

Apart from students' different learning abilities, Lai et al. [23] emphasized that the quality of online platform which teachers uploaded also counted a lot. The higher the platform quality, the higher students' autonomous motivation would be. For example, if the videos online were interesting enough, more students would be attracted and willing to watch. In this study, the teacher improved the quality of online platform not only by uploading good online resources, but also by clarifying the requirements of the preview tasks while designing autonomous learning assignment. As mentioned in *3.1 students' preferences for learning styles* part, many students preferred the way that the teacher had explained and guided students before they finished their preview tasks, and therefore, a concrete mind-map framework of each module's key and difficult points was provided to guide students' self-learning in the modified model (Fig. 2). Furthermore, the teacher added some personalized online and offline tutoring and guidance after class to help students understand preview tasks. The above factors were considered and integrated into the modified model (Fig. 2).

Additionally, the course design had influences on students' learning confidence, autonomous motivation and class engagement to a large extent [24]. Lai [25] also suggested a group-based flipped learning context because students would perform higher class engagement when they were involved in high group or peer interaction, and moderate task difficulty. This study involved in group marketing plan as a preview task.

The results in Table 3 showed that preview tasks before class could help to improve students' 'interpretation' and 'analysis' abilities. The main reason might be that most Chinese students received exam-oriented education which was a kind of indoctrinated education before entering the university. The exam-oriented education was passive and required less abilities of active exploration and independent thinking. Therefore, control groups relied more on the teacher's lectures in class, and neglected preview tasks. Meanwhile, experimental groups were more accustomed to self-learning and preview tasks under the flipped classroom approach. Completing preview tasks individually or through group exchanges, students could not only promote their divergent thinking, but also cultivate and stimulate the awareness of team communication and cooperation. At the same time, in the process of self-learning and group discussions, students needed to formulate a plan on the premise of fully understanding the task of the marketing plan and analyze various elements in the tasks, which could improve students' abilities of 'interpretation' and 'analysis'.

To conclude, before class, online resources should be good and clear enough to guide students, for example, a concrete mind map of framework on each module's key and difficult points should be provided for students' reference. The teacher also took individual differences and students' different learning abilities into account, and assigned optional tasks with difficulty gradient for students to choose. In the process of finishing preview tasks, students would understand the curriculum framework and knowledge, clarify key theoretical concepts, and form their own understanding and analysis of preview task problems through communication with group members. Thus, students' abilities of 'interpretation' and 'analysis' could be improved.

### 4.2 In class activities enhancing students' 'inference', 'evaluation' and 'explanation' abilities

Through the comparison between both practices shown in Table 4, three interesting findings could be discussed further as follows.

Firstly, 17 students in control classes and 10 students in experimental classes disagreed or strongly disagreed that the in-class test at the beginning of the class could test their understanding of the theoretical concepts and preview effects. The main reason might relate to the fact that they did not fully understand online resources and preview tasks, so the students couldn't do well at in-class tests. In order to solve the problem, in the second practice, the teacher commented and explained in time after the students had finished in-class tests, and helped students sort out key and difficult points in various ways such as group discussions, situational analysis, and case analysis, etc. The teacher could implement classroom interaction with the help of some technologies. Teachers with good interaction skills could arise students' interests of learning and participation in flipped classroom [23]. In order to explain and guide students to fully understand the course contents, the teacher also let students form a group to discuss cases related to the theories and analyze the cases by drawing a group mind map which could help students clarify the context of the case and give them a clearer understanding of the course contents (with over 96 % respondents agreed or strongly agreed on the effect of mind map activities shown in Table 4). At the same time, the teacher could stimulate students' enthusiasm for participation, creating a classroom atmosphere of democratic equality and mutual respect between the teacher and students, because only in this way could students dare to speak freely, raise questions and refute ideas, thus promoting the influx of new ideas and triggering more thinking and discussions among students. With the above improvements, 92 % respondents (69 students) approved or strongly approved of the effectiveness of the in-class tests in both cohorts after the second practice.

Secondly, both first and second practices showed that generally experimental groups achieved a higher sense of identity for the enhancement of several critical thinking abilities through the process of group marketing plan presentation than control groups did. The main reason was likely be that experimental groups had already integrated weekly course contents into the marketing plan and demonstrated the part of marketing plan week by week before the integrated marketing plan displayed in the last two teaching weeks. In every week's seminars, presenting groups showed the group marketing plan, and audience groups made interactive comments. During this process, many contents of the marketing plan in presenting groups, such as the understanding of the marketing concepts or the feasibility of the plan, would be questioned after being evaluated by audience groups. Audience groups would put forward many alternative plans that presenting groups did not think of, and stated the reasons why their alternative plans were better. Presenting groups would also fully and clearly explain and defend the marketing plan of their own groups based on the doubts and other views of the peers. This process also cultivated the critical thinking abilities of students who were good at listening to others' words and open to accept, and promoted students' in-depth understanding of the course contents. This process adopted a peer-review approach. Lin et al. [26] developed an online interactive peer-review approach, different from the conventional peer-review approach with assessors giving comments only and without assessees responding to the comments. The online interactive peer-review approach developed by Lin et al. involved in a two-way interaction between assessors and assessees. In this way, assessees could reflect and give feedback to assessors' comments. In the meanwhile, assessors could understand assessees' thinking fully and rejudge their ratings, thus really helping assessees. The research found that this online interactive peer-review approach could not only greatly strengthen students' knowledge and abilities to deal with specific comments, but also facilitate their critical thinking and reflective thinking abilities [26]. However, Zhang et al. [16] regarded not all groups' critical thinking abilities were similar. For high-score groups, students' critical thinking abilities could reach evaluation level instead of staying at understanding level, while for low-score groups, students' critical thinking abilities had more connections between understanding and analyzing levels. In this study, apart from peer-review, the teacher could also know students' misunderstandings on the course contents in this process, made comments on students' marketing plan and peer evaluation, or guided students to rethink and discuss the contents in question. The teacher's comments and explanations were supplemented by case analysis, group discussions, situational analysis and other diversified activities, so that students could fully grasp and apply the theories of marketing. Finally, students were able to think more alternatives, question various views, evaluate in many ways, explain by reasoning, and finally achieve the effects of better understanding of the course contents. In this process, students' abilities of 'inference', 'evaluation' and 'explanation' could be improved.

Thirdly, over 96 % respondents (72–74 students) of all groups in both first and second practices regarded that the mind map on the case analysis in class could help them clarify the case logic and context, and give them a clearer understanding of the course contents. The finding proved that the mind map contributed to sorting out the logical relationships between theories and practical applications. Therefore, students' comprehensive application abilities integrating understanding, analysis, evidence seeking, credibility proving, and explanation, etc. could be shown clearly in the mind map. Accordingly, students' capabilities for 'interpretation', 'analysis', 'inference', 'evaluation' and 'explanation' could also be shown.

In conclusion, in class, the teacher gave students timely guidance for students' misunderstandings in the in-class tests, and helped students sort out key and difficult points in various activities such as group discussions, situational analysis, and case analysis, etc. Different ways, such as group mind map analysis, could also be used to help students clarify the context of the case and acquire a clearer understanding of the course contents. In addition, the teacher focused on the interaction with students and created a classroom atmosphere of democratic equality and mutual respect between the teacher and students, because only in this way could students dare to speak freely, raise questions and refute ideas, thus promoting the influx of new ideas and triggering more thinking and discussions among students. Based on a sound classroom atmosphere, students gave group marketing plan presentation, did self-reflection and peer reviews, and thought of the teacher's comments in class. During the process of marketing plan presentation and evaluation, students were able to think more alternatives, question various views, evaluate in many ways, explain by reasoning, and finally achieve the effects of better understanding of the course contents. Therefore, students' abilities of 'inference', 'evaluation' and 'explanation' could be enhanced.

### 4.3 After class reflection positively affecting students' 'self-regulation' abilities

According to Table 5, participants who accepted flipped classroom approach were significantly more confident in their capabilities of rethinking profoundly of the course contents and agreed more with the view that the final group integrated marketing plan presentation was helpful to promoting their understanding and application of the *Marketing* course contents. In both practices, even though students in control classes attended *Marketing* seminars and learned 14 modules of the course, those students were still probably used to passive learning and lacked internalization of the course contents. In addition, experimental groups got 14 seminars' discussion opinions and modification guidance on marketing plan improvements. In this way, experimental groups could recheck, analyze and evaluate their understanding of the course contents, and corrected their metacognition. The self-reflection facilitates the processes of metacognition [27]. Therefore, students in control classes were unlikely to think of as many ideas on how to modify the marketing plan as students in experimental classes did. Meanwhile, the results indicated that students in experimental classes had better self-reflection abilities than students in control classes did, which meant that experimental classes tended to acquire better 'self-regulation' abilities.

In the modified model (Fig. 2), the grading criteria were improved by specifying contents, delivery, teamwork and overall evaluation. In this way, audience students could better revise their cognition of the course contents and tasks, actively consider the proposals of the marketing plan, and give objective evaluation to presenting groups, and thus improving their 'self-regulation' abilities.

Based on Table 7, six students in control groups stated that there were no opportunities to revise marketing plan at the last two seminars at the end of the first practice. Accordingly, more class activities and types of assignments would be allocated, and a written group marketing plan based on the modified group integrated marketing plan presentation was required to give students chances to modify the group marketing plan and reflect their work, and thus cultivating their 'self-regulation' abilities as well.

To sum up, after class, presenting groups would modify the group marketing plan, and report the modification parts in the next class. Students could ask for the teacher's personalized online and offline tutoring and guidance after class. In the next class, after presenting groups' reporting for the group integrated marketing plan, audience groups would give peers scores and explain the reasons by referring to the grading criteria with specification. Meanwhile, students should select excellent presenting groups on the spot, and the teacher would upload excellent marketing plan works on the online platform, which gave encouragement and confidence to the students in the excellent presenting groups, and cultivated peers' awareness of appreciation of others and 'self-regulation' abilities. In addition, a written group marketing plan based on the modified group integrated marketing plan presentation was required to give students chances to modify the group marketing plan, for the teacher could check whether students corrected their wrong understanding and improved the works, which could affect students' 'self-regulation' abilities. Furthermore, apart from the modified group integrated marketing plan presentation and written group marketing plan paper, students were also required to attend the final theoretical examination at the 17–18 examination weeks. Since students completed homework such as theoretical concept selection and judgment, short essay questions, discussion questions, and case analysis, which matched the question types in the final theoretical examination, students could choose or judge the theoretical concepts based on their understanding of the marketing theories and definitions, which could test their 'interpretation' skills. After students had understood the short essay questions and discussion questions, they could analyze the elements of the questions, find evidences and multiple possibilities to answer the questions, make a decision on the best answer, and finally explain the reasons for the answer comprehensively, thus proving students' 'analysis', 'inference', 'evaluation' and 'explanation' skills. Apart from all the five above critical thinking skills, case analysis required students' reflection on what they knew and comprehensively applied to the new case context, which required students' 'self-regulation' skills.

### 4.4 The flipped classroom approach having impacts on enhancing students' critical thinking skills

Some researchers compared flipped classroom teaching and traditional lectures among university or college level students in different countries and majors, and drew different conclusions. Some researchers approved of the relevance between flipped classroom approach and learning outcomes. For example, Ng [2] aimed at first-year students in nursing fundamental course at a Hong Kong community college and found that students could achieve better academic results in the final examination by accepting flipped classroom approach. Ito et al. [28] conducted a research among Japanese students majored in traditional medicine and also confirmed that students had better knowledge retention and academic performance, as well as attended class more often in flipped classes than in traditional classes.

Other researchers held opposite views. For example, Holma et al. [29] conducted the research in a statistics and epidemiology course at Oslo Metropolitan University. Participants involved in both classes had previous experience with both teaching approaches, and the research found that two groups achieved no big differences in exam grades, but preferred flipped classroom approach significantly. Elledge et al. [30] confirmed no significant academic outcomes in either group among medicine students. Surprisingly, Durrani et al. [11] drew the conclusion that the experimental group adopting gamified flipped classroom learning did not achieve course learning outcomes as well as the control group who accepting traditional classroom learning did due to perceived content relevance. The research also proved that it had some relevance of mixed learning approach rather than solely approach.

Refer to the statement 20 in Table 5, after the first practice, 61 respondents in control classes and 69 respondents in experimental classes respectively thought that they could do better at the final theoretical exams compared with other courses after learning the *Marketing* course. Generally, respondents in both cohorts were confident in their final theoretical exams compared with other courses. That probably because types of homework matched the final exam types, helping students to familiarize themselves with the theoretical examination to a certain extent. Furthermore, Chinese students were quite good at doing examination papers. Similar results were got after the second practice displayed in Table 5, with 60 control group students and 70 experimental group students approving or strongly approving of the statement 20. The above two results indicated that experimental groups who learned flipped classroom method believed that they had acquired a higher knowledge of the course than control groups who learned traditional teacher-centered approach did.

In this study, Table 8 compared students' results of the group integrated marketing plan and the theoretical examination, experimental groups after the second practice achieved an average score of 91.40 in the group integrated marketing plan, which got 6.19 marks higher than the first-practice experimental groups with 85.21 marks. Meanwhile, experimental groups after the second practice (93.88 marks) averagely scored 6.26 marks higher than experimental groups after the first practice (87.62 marks) did in the final theoretical examinations. The above two statistics showed that the modified flipped classroom approach was effective.

**Table 8:** Students' results of the group integrated marketing plan and the final theoretical examination.

Contents	Average score			
	The first practice		The second practice	
	Control classes (100 marks)	Experimental classes (100 marks)	Control classes (100 marks)	Experimental classes (100 marks)
The group integrated marketing plan	77.43	85.21	79.35	91.40
The final theoretical examination	82.56	87.62	83.98	93.88

In addition, according to Table 8, experimental groups achieved better academic results (about 5%–12 %) in both course assessments than control groups did, which proved that flipped classroom approach was effective and really had influences on enhancing students' critical thinking skills to some extent.

## 5 Implication of the study

A modified model of flipped classroom was put forward and tested effective to some extent in this study. The methods and procedures of this study can be duplicated and applied in *Marketing* courses. Designing a group marketing plan as an integrated task which combines all the modules in the *Marketing* course, students can be asked to adopt the flipped classroom approach by previewing each module themselves and finishing the particular part of the marketing plan in groups, then presenting and peer-evaluating the group marketing plan in class, finally reflecting and modifying the group marketing plan and reporting the modification in the next class. In this way, students can benefit from the appropriate learning method. At the same time, their learning efficiency will be enhanced and learning interests will be motivated.

Furthermore, the pedagogy introduced in this research can be applied to more other courses at different levels, especially business courses, for example *Management*, *Marketing Research*, *Consumer Behavior*, etc. Since most business courses have their own curriculum systems, integrated group tasks can be designed based on the course. For example, management case studies can be analyzed in groups based on each *Management* module. Marketing research plans can be assigned as group surveys in *Marketing Research* course. Group consumer behavior reports on real buying situations can be allocated in *Consumer Behavior* course.

## 6 Conclusions

This study put forward a modified model of flipped classroom approach which could enhance students' critical thinking skills raised by Peter Facione. The critical thinking skills were 'interpretation', 'analysis', 'inference', 'evaluation', 'explanation' and 'self-regulation'. The activities involved in the modified model (Fig. 2) were tested effective. Preview tasks before class could improve students' 'interpretation' and 'analysis' abilities. In class activities could enhance students' 'inference', 'evaluation' and 'explanation' abilities. After class reflection positively affected students' 'self-regulation' abilities.

According to the results of group integrated marketing plans and theoretical examination papers at the end of the course, students in experimental classes after the second practice got better grades than those experimental groups after the first practice did, which proved the effectiveness of the modified model of activities used in the flipped classroom approach. In addition, experimental groups achieved better results in both course assessments than control groups did, since they had higher understanding on the theories and greater abilities for applying the theories to the real situation. The results proved that students could benefit from the flipped classroom approach and therefore enhancing their critical thinking skills to some extent.

## 7 Limitations, pedagogical implications and directions for future researches

There were some limitations in this research. First of all, the questionnaire used a 5-point Likert scale. Some respondents might not consider the statements deeply and chose all the same scale for all the statements, and thus leading to some invalid data and weakening the persuasiveness of the data. The second limitation was related to participants. Since the study was conducted in a private university in China, the participants in the private university could not be the representative of all public and private university students in China and overseas. Moreover, the participants involved 300 students from three majors, there might be some individual differences among the participants. Therefore, the learning effects and experimental results might not be duplicated. Last but not least, the study chose *Marketing* course as the case of the research, which seemed that not sufficient readers could benefit from the research.

However, the pedagogy introduced in this research can be applied to more other courses at different levels, especially business courses. Since most business courses have their own curriculum systems, integrated group tasks can be designed based on different courses. In the future research, great importance can be attached to other skill development based on the course or researches on effectiveness of course feedback and assessment. Attention can also be paid to integrating other aspects like sustainable development, social and cultural awareness, and interdisciplinary knowledge, etc. into the course. Furthermore, if the marketing plan can be implemented, students' abilities on putting knowledge into practice will be evaluated.

## Funding statements

1. This study was supported by the Chenguang Program of 10.13039/501100003024Shanghai Education Development Foundation and 10.13039/501100003395Shanghai Municipal Education Commission. The project name was 'Research on the Enhancement of Critical and Innovative Thinking of Private University Students by Flipped Classroom Teaching Approach'. Grant Number: 19CGB08 (Q20012.20.001G-2019).

2. This study was supported by 10.13039/501100003395Shanghai Municipal Education Commission, Shanghai Education and Health Work Committee of the Communist Party of China, and XIANDA College of Economics & Humanities 10.13039/501100011994Shanghai International Studies University. The project name was '*Marketing* Model Course'. Grant Number: 2022KC254 (A3111.23.0802.014).

3. This study was supported by XIANDA College of Economics & Humanities 10.13039/501100011994Shanghai International Studies University. The project name was 'Shanghai First-class Undergraduate Course Cultivation Project: *Marketing* Online and Offline Hybrid Course'. Grant Number: A3024.22.136.

## Declaration of ethics and interests

XIANDA College of Economics & Humanities Shanghai International Studies University was the ethics committee approving the experiments of the study, with approval numbers Q20012.20.001G-2019, A3111.23.0802.014, and A3024.22.136.

## Data availability

In order to protect the anonymity of the participants in the study, the data that has been used is confidential and not publicly available.

## CRediT authorship contribution statement

**Yan Ma:** Writing – original draft, Writing – review & editing, Conceptualization, Data curation, Formal analysis, Funding acquisition, Investigation, Methodology, Resources, Validation, Visualization.

## Declaration of competing interest

The author declare that they have no known competing financial interests or personal relationships that could have appeared to influence the work reported in this paper.

## Figures and Tables

**Figure:**
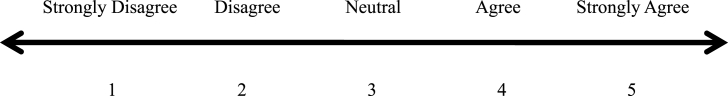


**Figure:**
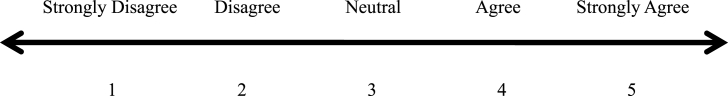


## Appendix 1

### The Questionnaire for *Marketing* Course

(The original version)

To what extent do you agree or disagree in terms of the 24 statements below:

Please choose from 1 to 5.

### Students' Preferences for Learning styles

1. I like self-learning first, and then the teacher tests the learning effects in class. The teacher just explains the problems reflected on the tests without teaching the course contents.
2. I like self-learning by finishing the preview tasks, and then present the preview effects in class. The peers and the teacher comments on the preview tasks. The teacher explains the problems reflected on the students' preview tasks without teaching the course contents.
3. I hope that the teacher will explain and guide me before I finish my preview tasks, and then present the preview effects in class. The peers and the teacher comments on the preview tasks. The teacher explains the problems reflected on the students' preview tasks without teaching the course contents.
4. I like the traditional way of learning. The teacher gives lectures in class and I learn instead of finishing the preview tasks and presenting the works in class.

### Students' Feedback on Preview Tasks Before Class

5. The assignment requirements for the preview tasks are clear, and I can fully understand how to do the tasks.
6. The online resources can guide me to learn independently, and I can fully understand how to do the preview tasks.
7. In the process of doing preview tasks, I can fully understand key theoretical concepts of the course.
8. In the process of doing preview tasks, I can identify the problems of the preview tasks.
9. Through the exchange of ideas in groups, I can form my own analysis and understanding of the preview tasks.
10. Through the exchange of ideas in groups, the group members can discuss and analyze together to promote my understanding of the course contents.

### Students' Feedback on Presentation and Peer Evaluation in Class

11. The in-class test at the beginning of the class can test my understanding of the theoretical concepts and preview effects.
12. As a member of the audience group, by watching and listening to other groups' marketing plan presentations in class, I can conceive many alternative plans that the presenting groups did not think of, and I can state the reasons why my alternative plans are better.
13. As a member of the audience group, by watching and listening to other groups' marketing plan presentations in class, I can acquire better understanding of the course contents through the process of questioning and judging the presenting groups' presentations.
14. As a member of the presenting group, I can gain a lot and promote my understanding of the course contents through peer reviews and the teacher's comments.
15. As a member of the presenting group, I can defend our group's opinions and reasoning fully and clearly.
16. The mind map on the case analysis in class can help me clarify the case logic and context, and give me a clearer understanding of the course contents.

### Students' ReDflection and Evaluation Feedback After Class

17.Through reflection after class and reporting in the next class, the process of improving and revising group marketing plan can let me rethink profoundly of the course contents and promote my understanding and application of this part of the course contents, thus increasing the sense of achievement.
18. I think the peer assessment after the reflection presentation is objective and can make me gain a lot.
19. I think the marketing plan presentation evaluation is very important, and it is necessary to publish the presentation grading criteria in advance.
20. I think the presentation grading criteria is reasonable and can guide me to make a good presentation.
21. I think that showing excellent marketing plans online after class can improve my understanding of the course contents by appreciating peers' model works.
22. I think that the final group integrated marketing plan presentation is helpful to my understanding and application of the *Marketing* course.
23. I think that homework including theoretical concept selection and judgment, short essay questions, discussion questions, and case analysis can help me do better at the final theoretical exams.
24. After learning the course, I can do better at the final theoretical exams compared with other courses.

### Multiple Choice

25. What are you satisfied with this course______________?

A. Teaching method
B. Task requirements and varieties
C. Online and offline resources
D. In-class activities
E. Homework load
E. Evaluation and assessment
F. Teaching feedback and guidance
G. Class atmosphere
H. Interaction between the teacher and students
I. Others, please specify _______________.

## Appendix 2

### The Questionnaire for *Marketing* Course

(The revised and applied version)

Students' Preferences for Learning styles.

Which learning style do you like best? Please choose your favorite one.

A. I like self-learning first, and then the teacher tests the learning effects in class. The teacher just explains the problems reflected on the tests without teaching the course contents.
B. I like self-learning by finishing the preview tasks, and then present the preview effects in class. The peers and the teacher comments on the preview tasks. The teacher explains the problems reflected on the students' preview tasks without teaching the course contents.
C. I hope that the teacher will explain and guide me before I finish my preview tasks, and then present the preview effects in class. The peers and the teacher comments on the preview tasks. The teacher explains the problems reflected on the students' preview tasks without teaching the course contents.
D. I like the traditional way of learning. The teacher gives lectures in class and I learn instead of finishing the preview tasks and presenting the works in class.

To what extent do you agree or disagree in terms of the 20 statements below:Please choose from 1 to 5.

### Students' Feedback on Preview Tasks Before Class

1. The assignment requirements for the preview tasks are clear, and I can fully understand how to do the tasks.
2. The online resources can guide me to learn independently, and I can fully understand how to do the preview tasks.
3. In the process of doing preview tasks, I can fully understand key theoretical concepts of the course.
4. In the process of doing preview tasks, I can identify the problems of the preview tasks.
5. Through the exchange of ideas in groups, I can form my own analysis and understanding of the preview tasks.
6. Through the exchange of ideas in groups, the group members can discuss and analyze together to promote my understanding of the course contents.

### Students' Feedback on Presentation and Peer Evaluation in Class

7. The in-class test at the beginning of the class can test my understanding of the theoretical concepts and preview effects.
8. As a member of the audience group, by watching and listening to other groups' marketing plan presentations in class, I can conceive many alternative plans that the presenting groups did not think of, and I can state the reasons why my alternative plans are better.
9. As a member of the audience group, by watching and listening to other groups' marketing plan presentations in class, I can acquire better understanding of the course contents through the process of questioning and judging the presenting groups' presentations.
10. As a member of the presenting group, I can gain a lot and promote my understanding of the course contents through peer reviews and the teacher's comments.
11. As a member of the presenting group, I can defend our group's opinions and reasoning fully and clearly.
12. The mind map on the case analysis in class can help me clarify the case logic and context, and give me a clearer understanding of the course contents.

### Students' Reflection and Evaluation Feedback After Class

13. Through reflection after class and reporting in the next class, the process of improving and revising group marketing plan can let me rethink profoundly of the course contents and promote my understanding and application of this part of the course contents, thus increasing the sense of achievement.
14. I think the peer assessment after the reflection presentation is objective and can make me gain a lot.
15. I think the marketing plan presentation evaluation is very important, and it is necessary to publish the presentation grading criteria in advance.
16. I think the presentation grading criteria is reasonable and can guide me to make a good presentation.
17. I think that showing excellent marketing plans online after class can improve my understanding of the course contents by appreciating peers' model works.
18. I think that the final group integrated marketing plan presentation is helpful to my understanding and application of the *Marketing* course.
19. I think that homework including theoretical concept selection and judgment, short essay questions, discussion questions, and case analysis can help me do better at the final theoretical exams.
20. After learning the course, I can do better at the final theoretical exams compared with other courses.

### Open Question

What are you satisfied and dissatisfied with this course? Please specify.

(You can refer to the teaching method, task requirements and varieties, online and offline resources, in-class activities, homework load, evaluation and assessment, teaching feedback and guidance, class atmosphere, and interaction between the teacher and students, etc.)
